# Changes in bilateral tear film and corneal nerve stability in patients with unilateral neurotrophic keratitis

**DOI:** 10.3389/fmed.2025.1531673

**Published:** 2025-05-14

**Authors:** Fan Wang, Yue Li, Zhaowei Zhang, Qiuchen Lu, Cancan Shi, Xiaofan Yu, Fen Hu, Mingxin Li, Jianxin Guo, Zhenhao Zhang, He Wang

**Affiliations:** ^1^Department of Ophthalmology, The Affiliated Hospital of Xuzhou Medical University, Xuzhou, China; ^2^Xuzhou Medical University, Xuzhou, China; ^3^Research Center, Shanghai University of Medicine and Health Sciences Affiliated Zhoupu Hospital, Shanghai, China

**Keywords:** neurotrophic keratitis, confocal microscopy, corneal sensitivity, tear film, ocular surface

## Abstract

**Objective:**

This study aimed to examine the bilateral stability of the tear film in patients with unilateral neurotrophic keratitis and to observe changes in corneal nerve and immune cells under confocal microscopy.

**Methods:**

A prospective cross-sectional study included 63 patients with confirmed neurotrophic keratitis (NK) and 40 normal controls of similar sex and age. NK patients were divided into stage 1 and stage 2 based on the severity of the disease. Tear meniscus height (TMH), first non-invasive tear film break-up time (NIBUT-f), and corneal fluorescein staining were assessed. Corneal sensitivity was assessed using a Cochet-Bonnet esthesiometer. The corneal subbasal nerve plexus (SNP) and dendritic cells (DCs) were imaged using *in vivo* confocal microscopy (IVCM), and the SNP was analyzed using the fully automated corneal nerve analysis software “ACCmetric.”

**Results:**

Eyes with NK stage 2 showed worse performance in TMH, NIBUT-f, corneal fluorescein staining score, corneal sensitivity examination, SNP parameters, and DC density compared to NK stage 1 (all *p* < 0.001). The contralateral eyes of NK patients had significantly shorter NIBUT-f and higher dendritic cell density than controls (*p* < 0.001).

**Conclusion:**

The contralateral eyes of NK patients are more prone to dry eye signs than those of normal subjects and should be monitored and treated promptly. Increased dendritic cell numbers in the contralateral eyes of NK patients suggest bilateral immune alterations in unilateral disease.

## 1 Introduction

Neurotrophic keratitis (NK) is a degenerative condition of the cornea caused by trigeminal nerve damage (1). Common causes include herpes virus infections, injuries from brain and eye surgery, and diabetes, which can result in dry eye, corneal epithelial defects, and corneal ulcers. NK is a relatively rare disease with an incidence of approximately 1.6 per 10,000, including approximately 6% of patients with herpetic keratitis, 12.8% of patients with varicella zoster keratitis, and 2.8% of patients with trigeminal neuralgia who undergo surgery, among other causes (2–5).

The diagnosis of NK is based on a medical history, slit lamp examination, and corneal sensitivity. In recent years, the advent of IVCM has provided new ideas for the diagnosis of NK (6). The corneal sensory nerve originates from the ophthalmic branch of the trigeminal nerve and is one of the most nerve-rich tissues in the body. The corneal sensory nerve responds to damage to the ocular surface by triggering symptoms of pain and irritation in the eye and by triggering protective reflexes such as blinking and tearing. Corneal nerve fibers also release neuromediators that provide nutritional support to ocular surface tissues, stimulate wound healing, and maintain the anatomical integrity of the cornea (7, 8). Moreover, the ophthalmic branch of the trigeminal nerve plays an important role in maintaining tear film stability by regulating eyelid movements and tear secretion from the lacrimal gland, goblet cell, and meibomian gland (9). Damage of varying degrees from the trigeminal nerve to the corneal nerve endings in the plexus can lead to hyperalgesia and imbalance in tear film stability, which in turn can lead to corneal epithelial damage and the development of neurotrophic keratitis. In severe cases, the exposed stroma is susceptible to enzymatic degradation, leading to melting and eventual perforation (10).

The impact of corneal nerve damage on the ocular surface in patients with unilateral NK is well recognized (11), while changes in tear film stability in the contralateral eye are often overlooked. However, patients with unilateral NK often complain of eye dryness, foreign body sensation, and burning sensation in their contralateral eye. Therefore, it is imperative for clinicians to pay attention to the ocular surface condition of bilateral eyes.

The aim of this study was to observe tear film stability and corneal nerve condition in eyes with different stages of unilateral NK and in the contralateral eye, using tear meniscus height, tear film break-up time, sodium corneal fluorescein staining, corneal sensitivity examination, and IVCM, along with fully automated corneal nerve analysis software.

## 2 Materials and methods

This is a prospective, cross-sectional, controlled, single-blind study. According to the severity of the condition, Mackie divides NK into three stages, Hyperplasia and irregularity of the corneal epithelium led to a cloudy appearance of the epithelium in NK stage 1 regardless of whether superficial punctate keratopathology is present; NK stage 2 is characterized by a persistent epithelial defect on the cornea, located mainly in the superior paracentral region of the cornea. An oval or circular shape with smooth edges and rolled corners is caused by impaired healing. NK stage 3 progresses to involve the stroma and is characterized by the development of a corneal ulcer, which may lead to perforation and/or stromal melting (12). Sixty-three patients with unilateral stage 1 or stage 2 NK were recruited from the Neurosurgery Department of the Affiliated Hospital of Xuzhou Medical University. These patients had previously undergone neurosurgical procedures for acoustic neuroma, trigeminal neuralgia, or tumors. All surgeries were performed by the same group of experienced doctors.

All affected eyes and 30 randomly selected contralateral eyes were studied, with 40 normal volunteers all using their right eyes as a control group. This study was conducted by the Declaration of Helsinki and approved by the Ethics Committee of Xuzhou Medical University. Written informed consent was obtained from all participants. A detailed medical history was taken from the patients. The diagnosis of NK was made by the presence of a typical clinical image on slit lamp examination, associated with reduced or absent corneal sensitivity. The exclusion criteria included NK stage 3, diabetes mellitus, autoimmune disease, history of ocular surgery, history of ocular trauma, use of contact lenses, and patients already receiving treatment other than artificial tears. Exclusions also applied to patients receiving systemic corticosteroids at the time of the examination.

The tear meniscus height (TMH) and the first non-invasive tear film break-up time (NIBUT-f) were measured using an ocular surface comprehensive analyzer (DED-1 L, Kanghua, Chongqing, China). (1) TMH test: the height of the lowermost tear trough at the center of the pupil is measured with a self-contained scale. Normal value >0.2 mm. (2) NIBUT-f: The Placido ring image is projected onto the patient’s cornea using an infrared light source. The patient is asked to blink twice before the system enters the automatic detection mode. The system then automatically obtains the results, repeats the measurement three times, and calculates the average value. Normal value: >10s.

Sodium fluorescein staining of the cornea: the sodium fluorescein test paper is moistened with one drop of saline, making sure that the edge of the test paper is moistened, and then the excess dye is gently shaken off. The patient’s lower lid is gently turned outward, and the moistened test strip is gently placed in contact with the conjunctival sac of the lower lid to mix the fluorescein tears. The patient is asked to blink three to five times naturally and then to gaze forward. A rating between 0 and 5 according to the Oxford scale (13).

Patients and controls were evaluated for central corneal sensitivity with the Cochet-Bonnet esthesiometer. By pressing a retractable 6-cm-long monofilament nylon thread against the anterior corneal surface, this test mechanically stimulates the corneal nerves. If a positive response is not obtained, the length is reduced in increments of 1.0 cm. Once a positive response is obtained, the thread is advanced by 0.5 cm, and the longest filament length resulting in a positive response is considered the corneal sensitivity threshold (14).

A corneal confocal microscope (HRT3, Heidelberg, Germany) was used to scan the central cornea of all NK patients and the right eye of normal controls. Topical anesthesia with oxybuprocaine hydrochloride was applied to the eye, followed by one drop of carbomer eye drops. The subject kept looking forward, while the operator adjusted the chin and forehead supports to align the subject’s eyes vertically with the microscope probe, and adjusted the distance between the cornea and objective lens to 5 to 10 mm. Continuous confocal tomography was used to obtain images of the various layers of tissues and cells in the central cornea. Using the sequence mode, 100 digital images were recorded per sequence at a rate of 3 frames per second. An average of six to eight sequence scans of non-overlapping areas were recorded in the corneal subbasal nerve plexus (SNP) layer of the 3-mm central cornea. An image of the nerve plexus was taken at a depth of 50 to 80 μm, using a frame size of 400 × 400 μm.

IVCM examinations were conducted by experienced examiners. By using a masked examiner, our six most representative images of corneal SNP were selected based on the optimal contrast, the presence of in-focus nerves, and the absence of motion artifacts. The following metrics were calculated for each IVCM image using the fully automated image analysis software ACCMetrics (MA Dabbah, Imaging Science and Biomedical Engineering, Manchester, UK): corneal nerve fiber density (CNFD) (n/mm^2^), corneal nerve branch density (CNBD) (n/mm^2^), corneal nerve fiber length (CNFL) (mm/mm^2^), corneal nerve total branch density (CTBD) (n/mm^2^), corneal nerve fiber area (CNFA) (mm^2^/mm^2^), corneal nerve fiber width (CNFW) (mm/mm^2^), and corneal nerve fractal dimension (CfracDim) (15). Dendriform cell quantification (cells/mm^2^) was performed using Cell Count software (Heidelberg Engineering GmbH) in the manual mode by identifying bright individual dendriform structures with cell bodies in each image at the level of basal epithelium or subbasal nerve plexus.

### 2.1 Statistical analysis

Statistical analysis was performed using SPSS 26.0 software (SPSS Inc., Chicago, Illinois, USA). Values are expressed as mean ± standard deviation and one-way ANOVA was used for the comparison of differences in normally distributed data, and the least significant difference method was used for further two-way comparisons between groups. The Kruskal–Wallis H-test was used to compare differences in non-normally distributed data and further two-way comparisons between groups were performed with the Bonferroni correction. The difference was considered statistically significant at a *p*-value of <0.05.

## 3 Results

The demographic data of NK patients and controls are shown in Table 1. There were no statistically significant differences between controls, NK stage 1, NK stage 2, or their contralateral eyes in terms of age (52.2 ± 9.8 vs. 49.5 ± 9.9 vs. 52.7 ± 8.7 vs. 48.5 ± 8.0 vs. 50.6 ± 8.5y, *p* = 0.287) or gender (21/19 vs. 17/20 vs. 14/12 vs. 17/20 vs. 14/12, *p* = 0.928).

**Table 1:** Demographic data of controls and patients with NK.

	controls	NK stage 1	NK stage 2	NK1’	NK2’	F	P
Sample size	40	37	26	37	26		
age	52.2 ± 9.8	49.5 ± 9.9	52.7 ± 8.7	48.5 ± 8.0	50.6 ± 8.5	1.263	0.287
Sex(male/female)	21/19	17/20	14/12	17/20	14/12	0.218	0.928

“NK1’”: the contralateral eyes of patients at NK stage 1; “NK2’”: the contralateral eyes of patients at NK stage 2.

**Figure 1:**
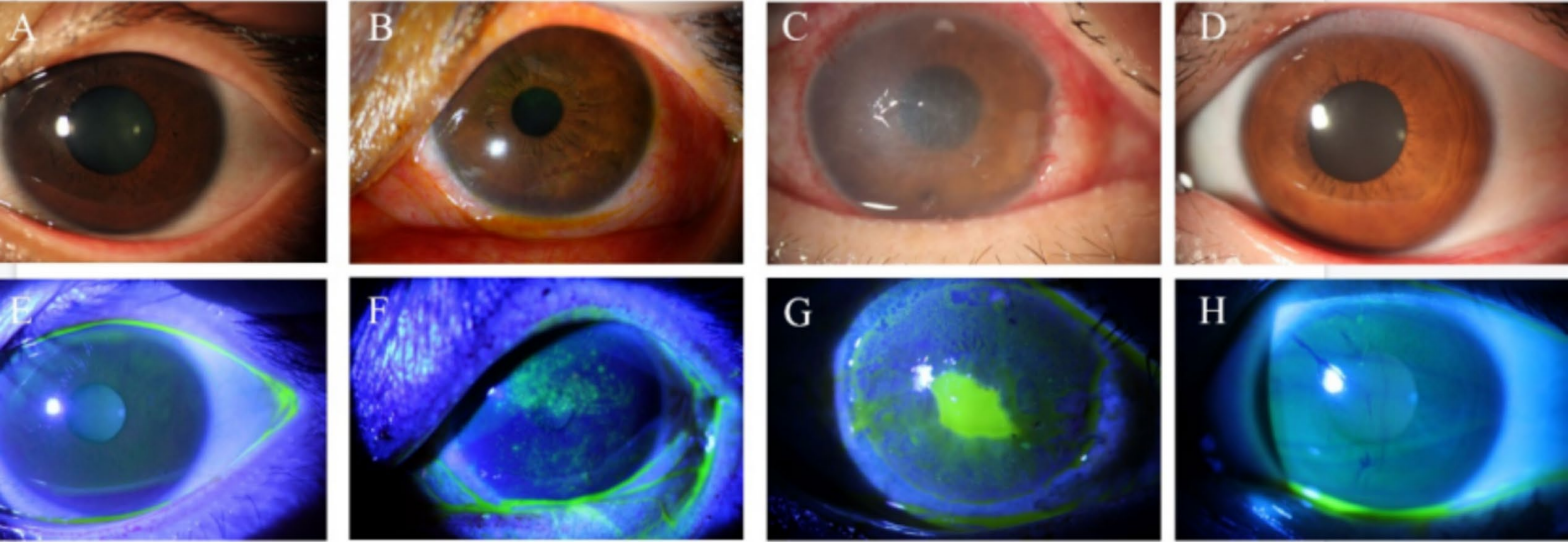
Slit lamp and corneal fluorescein staining images of controls and patients with NK. **(A)** Slit lamp examination in the control group. **(B)** Slit lamp examination at the NK stage 1. **(C)** Slit lamp examination at the NK stage 2. **(D)** Slit lamp examination in the contralateral eye. **(E)** Corneal fluorescein sodium staining in the control group. **(F)** Corneal fluorescein sodium staining in the NK stage 1. **(G)** Corneal fluorescein sodium staining at the NK stage 2. **(H)** Corneal fluorescein sodium staining in the contralateral eye.

**Figure 2:**
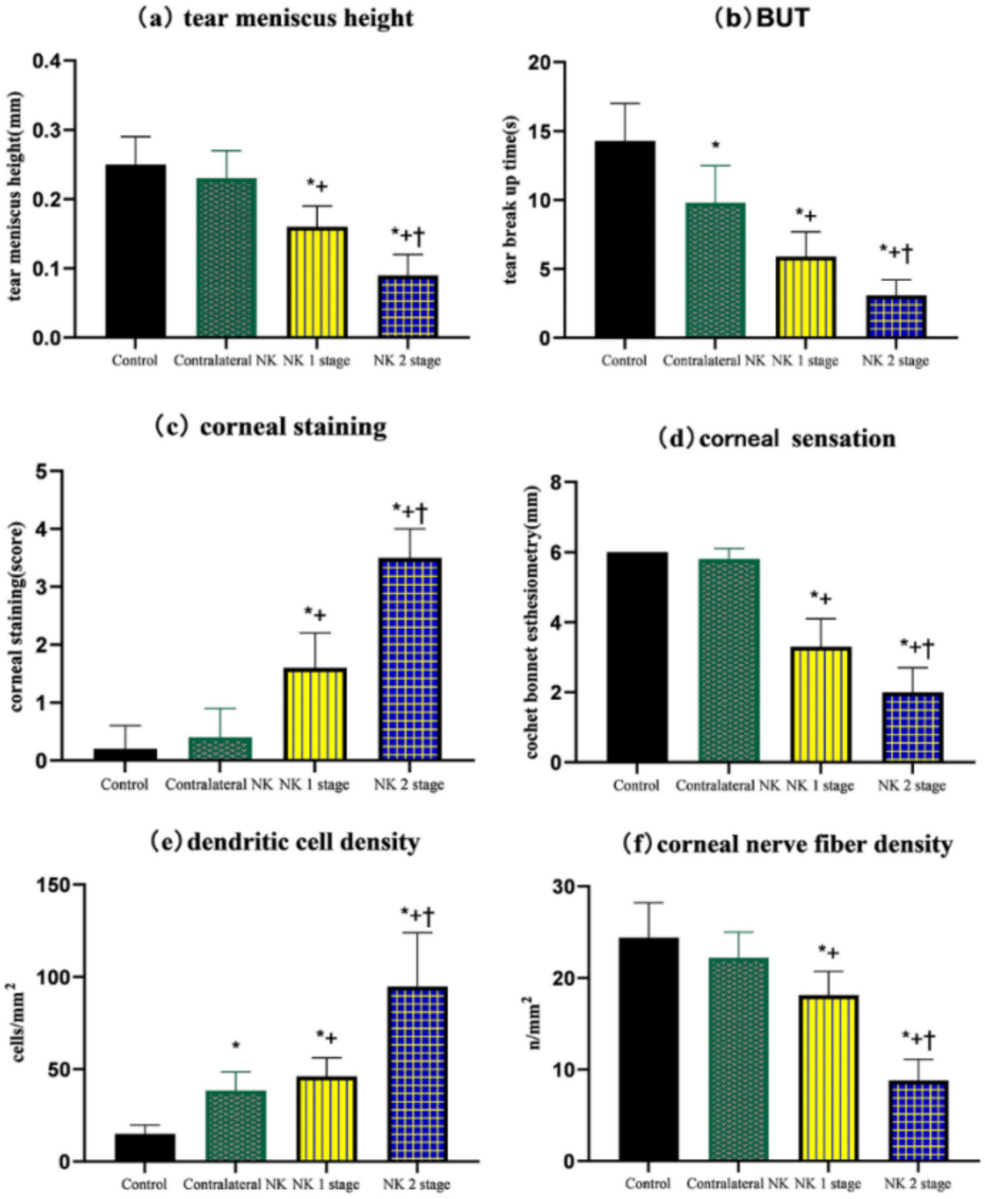
Comparison of TMH, NIBUT-f, corneal fluorescein staining, corneal sensation, dendritic cell density, and corneal nerve fiber density of controls and patients with NK. **(A)** TMH. **(B)** BUT. **(C)** Corneal staining. **(D)** Corneal sensation. **(E)** Dendritic cell density. **(F)** Corneal nerve fiber density. Values are expressed as mean ± standard deviation and one-way ANOVA was used for the comparison of differences. *p < 0.05.

The results of the ocular surface indicators examination are presented in Table 2. Eyes at the NK stage 2 exhibited worse TMH and NIBUT-f compared to eyes at the NK stage 1 (both *p* < 0.001). Additionally, there was a worse performance in TMH and NIBUT-f in the NK-affected eyes compared to the eyes of controls and the contralateral eyes (all *p* < 0.001). Interestingly, NIBUT-f was significantly lower in the contralateral eyes of NK patients than in the controls (*p* < 0.001). As shown in Figure 1, the eyes with NK2 had higher corneal fluorescein staining scores than those with NK1 (*p* = 0.002). The scores were also significantly higher in NK-affected eyes compared to the eyes of controls and the contralateral eyes (all *p* < 0.001). However, the difference in corneal fluorescein staining scores between the contralateral and control eyes was not significant (*p* = 0.344). The statistical charts of the ocular surface parameters for each group are shown in Figure 2. The results of the coneal perception examination are shown in Table 2 and Figure 2. Corneal sensitivity was significantly reduced in the eyes of the NK stage 2 compared to those of the NK stage 1 (*p* = 0.044). Corneal sensitivity was reduced in the contralateral eyes compared to the control group, with a non-significant difference (*p* = 0.427). Corneal sensitivity was significantly lower in NK-affected eyes compared to control and contralateral eyes (both *p* < 0.001).

**Table 2:** Ocular surface parameters of controls and patients with NK.

	controls	NK stage 1	NK stage 2	NK1’	NK2’	F	P
TMH	2.5 ± 0.4	1.6 ± 0.3	0.9 ± 0.3	2.3 ± 0.4	2.5 ± 0.3	117.8	<0.001***
NIBUT-f	14.3 ± 2.7	5.9 ± 1.8	3.1 ± 1.1	9.8 ± 2.7	9.4 ± 2.4	113.8	<0.001***
Corneal staining	0.2 ± 0.4	1.6 ± 0.6	3.5 ± 0.5	0.4 ± 0.5	0.6 ± 0.2	168.5	<0.001***
Corneal sensitivity	6.0 ± 0.1	3.3 ± 0.8	2.0 ± 0.7	5.8 ± 0.3	5.1 ± 0.7	343.1	<0.001***

“NK1’”: the contralateral eyes of patients at NK stage 1; “NK2’”: the contralateral eyes of patients at NK stage 2, ***p < 0.001.

**Figure 3:**
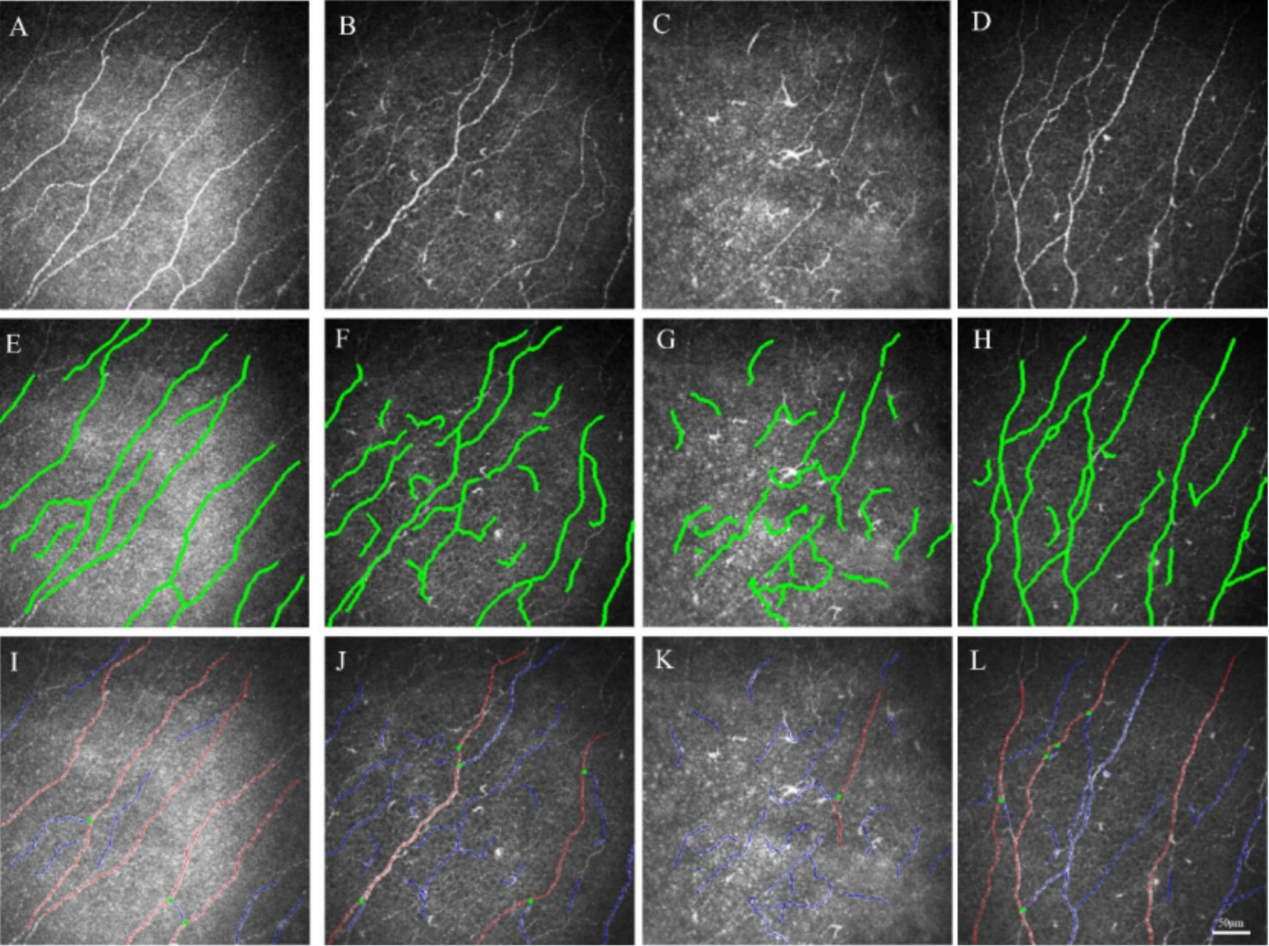
IVCM examination of controls and patients with NK and analysis of image results using ACCMetrics software. **(A)** *In vivo* confocal microscope scan of the subbasal nerve plexus in the control group. **(B)** *In vivo* confocal microscope scan of the subbasal nerve plexus at the NK stage 1. **(C)** *In vivo* confocal microscope scan of the subbasal nerve plexus at the NK stage 2. **(D)** *In vivo* confocal microscope scan of the subbasal nerve plexus in the contralateral eye. **(E,I)** Analysis of control SNP images using ACCMetrics software. **(F,J)** Analysis of NK stage 1 SNP images using ACCMetrics software. **(G,K)** Analysis of NK stage 2 SNP images using ACCMetrics software. **(H,I)** Analysis of contralateral eye SNP images using ACCMetrics software.

As shown in Table 3 and Figure 3, DC density was significantly higher in the eyes at the NK stage 2 than in the NK stage 1, contralateral eyes, and controls (all *p* < 0.001). Surprisingly, the density of DCs in the contralateral eyes of NK also significantly exceeded that of the control group (*p* < 0.001).

**Table 3:** *In vivo* confocal microscopy parameters of controls and patients with NK.

	controls	NK stage 1	NK stage 2	NK1’	NK2’	F	P
CNFD	24.4 ± 3.8	18.1 ± 2.6	8.8 ± 2.3	22.2 ± 2.8	25.2 ± 3.1	144.6	<0.001***
CNBD	31.2 ± 5.6	20.9 ± 3.8	9.9 ± 3.2	27.5 ± 4.7	25.5 ± 8.7	81.5	<0.001***
CNFL	13.7 ± 1.8	10.3 ± 1.5	7.3 ± 1.2	13.6 ± 1.8	16.6 ± 2.8	103.9	<0.001***
CTBD	46.4 ± 10.5	30.4 ± 7.3	16.3 ± 3.9	43.9 ± 10.0	32.4 ± 11.0	56.7	<0.001***
CNFA	0.006 ± 0.002	0.004 ± 0.001	0.002 ± 0.001	0.007 ± 0.002	0.004 ± 0.003	25.7	<0.001***
CNFW	0.254 ± 0.004	0.188 ± 0.004	0.008 ± 0.002	0.246 ± 0.004	0.196 ± 0.007	14890.9	<0.001***
CFracDim	1.589 ± 0.095	1.339 ± 0.091	0.891 ± 0.193	1.551 ± 0.118	2.001 ± 0.088	319.9	<0.001***
DCD	15.0 ± 4.8	46.1 ± 10.0	94.8 ± 29.0	38.4 ± 10.1	40.4 ± 8.2	139.0	<0.001***

“NK1’”: the contralateral eyes of patients at the NK stage 1; “NK2’”: the contralateral eyes of patients at the NK stage 2, ***p < 0.001.

**Figure 4:**
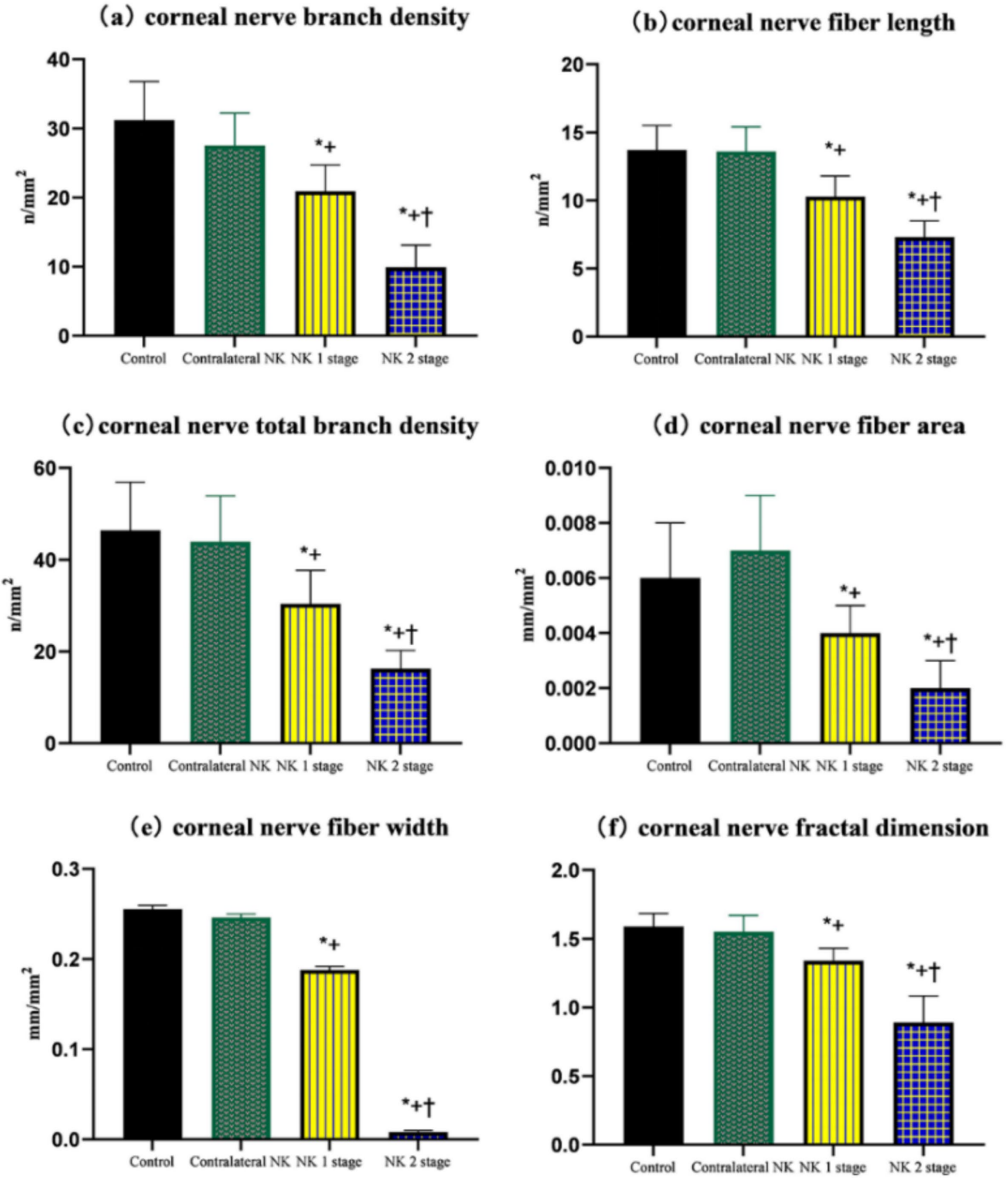
Comparison of corneal nerve branch density, corneal nerve fiber length, total corneal nerve branch density, corneal nerve fiber area, corneal nerve fiber width, and corneal nerve fractal dimension of controls and patients with NK. **(A)** Corneal nerve branch density. **(B)** Corneal nerve fiber length. **(C)** Total corneal nerve branch density. **(D)** Corneal nerve fiber area. **(E)** Corneal nerve fiber width. **(F)** Corneal nerve fractal dimension. Values are expressed as mean ± standard deviation and one-way ANOVA was used for the comparison of differences. *p < 0.05.

The results of SNP analysis under IVCM examination are shown in Table 3 and Figure 4. ACCMetrics analysis indicated that CNFD, CNBD, CNFL, CTBD, CNFA, CNFW, and CNFrD were significantly reduced in NK2 compared to NK1 (all *p* < 0.001). CNFD, CNBD, CNFL, CTBD, CNFA, CNFW, and CNFrD were significantly reduced in NK-affected eyes compared to contralateral eyes and controls (all *p* < 0.001). There was no significant difference in CNFD, CNBD, CNFL, CTBD, CNFA, CNFW, and CNFrD in the contralateral eyes of NK patients compared to the control group (all *p* > 0.05).

## 4 Discussion

In line with previous studies, our study showed that NK patients showed significant abnormalities in tear film stability, corneal fluorescein staining scores, corneal perception, and corneal nerve parameters compared to the control group (16, 17). In addition, we found that patients with NK stage 2 showed not only a significant difference in slit lamp examination compared to NK stage 1 but also a worse performance in terms of IVCM and tear film stability, suggesting that IVCM can be used as an important indicator of the severity of the disease. The decline in the morphology and function of corneal nerves often leads to varying degrees of corneal epithelial injury, probably due to the trophic effect of the corneal nerve on the corneal epithelium, while corneal epithelial and corneal cells affect nerve fiber survival, differentiation, and maturation through the release of neuropeptides, neurotrophins, and growth factors such as nerve growth factor (NGF), neurotrophic factor 3 (NT-3), nerve growth factor 4/5 (NT-4), epidermal growth factor (EGF), brain-derived neurotrophic factor (BDNF), ciliary neurotrophic factor, and glial cell-derived neurotrophic factor (GDNF) (18–21). Corneal epithelial injury leads to the exposure of the subepithelial nerve plexus, infiltration, and recruitment of inflammatory cells, release of inflammatory factors, and oxidative stress. These, in turn, can affect the normal function of the corneal nerves. Therefore, the course of NK should be a complex process in which the corneal nerve and corneal epithelium interact.

Innate and adaptive immunity are sentinels of dendritic cells, which are bone marrow-derived antigen-presenting cells. Infectious and inflammatory mediators activate antigen-specific DCs that migrate to secondary lymphoid organs via a chemokine gradient to initiate cell-mediated immunity by activating naive T cells (CD4+ and CD8+) and B cells (22, 23). The cornea is an immune-privileged tissue *in vivo.* Streilein et al. reported that ocular immune privilege is under neural control. The anterior chamber of the eye contains immunosuppressive neuropeptides. When the corneal nerve is damaged, the tissue surrounding the anterior chamber stops secreting these immunosuppressive factors, anterior chamber-associated immune deviation (ACAID) fails, and corneal immune cells increase (24, 25). In addition, we found an increased density of dendritic cells in the contralateral eye of NK patients compared to controls by IVCM, suggesting an inflammatory response in the contralateral eye, but the exact mechanism of this is unclear.

Tear secretion is mainly dependent on nerve stimulation. In NK patients, nerve damage reduces tear secretion and tear film stability, which in turn causes abnormal epithelial cell metabolism and epithelial damage, which may be one of the mechanisms of NK pathogenesis (26). In this study, we observed a significant reduction in tear river height and tear film break-up time in NK-affected eyes, and interestingly, the contralateral eye also showed a shortened tear film break-up time, probably due to corneal nerve damage in NK-affected eyes, reduced blink frequency, increased incomplete blink rate, and impaired tear film renewal (11). Jordan A. et al. found that local anesthetic drops in rabbit eyes reduced tearing by 75% and bilateral blinking by approximately 30%, and that tear film renewal was largely dependent on blinking action (27). Whether the increased DC density in the contralateral eye is a bilateral immune response to monocular nerve damage or a decrease in tear film stability in the contralateral eye, with signs of dry eye and a consequent inflammatory response, needs further study to confirm.

Unlike previous studies, this study did not find significant differences in contralateral corneal nerve parameters, such as CNFD, CNBD, CNFL, CTBD, CNFA, CNFW, and CNFrD compared to the control group. Possible reasons for this include the fact that the software used for corneal nerve analysis in previous studies was mostly semi-automatic, and although there was a high level of agreement between fully automatic and semi-automatic analysis software in terms of analyzing SNPs, there were differences in some indicators, such as CNFrD (28–30). Moreover, some studies have included patients with NK who had either herpes simplex keratitis or herpes zoster keratitis. Since viral infections can latently affect the trigeminal ganglion or corneal nerves, these patients may exhibit potential pathological changes in the corneal nerves, making it challenging to categorize them as “unilateral” NK patients. In this study, we included subjects with NK who underwent unilateral trigeminal nerve surgery. The trigeminal nerve in the contralateral eye was not affected, making the experimental results appear more reliable. The age of the patients included in this study was higher than before, and previous studies have confirmed that corneal nerve parameters may also change with age (31, 32).

The present study has the following limitations: First, we conducted only a cross-sectional study, which lacked a time-series analysis to evaluate the changes in the ocular surface condition and neural function of NK patients over time. Second, the sample size included in this study is limited, necessitating multi-center or larger sample studies to further validate the reliability of the experimental results. Third, while the study observed the presence of DC recruitment and changes in the tear film in the contralateral eye of NK patients, the causal relationship between the two remains to be further analyzed.

In summary, this study evaluated the ocular surface condition and changes in the corneal nerves of both eyes in patients with unilateral NK. It was found that, compared to the control group, NK patients exhibited lower tear film stability and impaired nerve function. The tear film abnormalities in the contralateral eye of patients with unilateral NK should be of particular concern. Clinicians should take measures to improve the quality of life and ocular surface health in NK patients.

## Data Availability

The raw data supporting the conclusions of this article will be made available by the authors, without undue reservation.
